# A Rasch analysis of the High Potential Trait Indicator: A South African sample

**DOI:** 10.4102/ajopa.v5i0.115

**Published:** 2023-02-08

**Authors:** David S. Semmelink, David J.F. Maree

**Affiliations:** 1 Department of Psychology, Faculty of Humanities, University of Pretoria, Pretoria, South Africa

**Keywords:** psychometric properties, high potential trait indicator (HPTi), Rasch model fit, person reliability, differential item functioning

## Abstract

The reliability and validity of the six traits comprising the High Potential Trait Indicator (HPTi) were evaluated using Rasch analysis. Focus was designated to the unidimensionality and local independence of each subscale; fit to the Rasch model; person reliability and separation; and differential item functioning (DIF). Secondary data, obtained from intellectual property rights holder Thomas International, were used for analysis with a sample of 1257 South African respondents. One of the six traits, *Curiosity* (0.73), was found to be reliable. Traits *Adjustment* (0.69) and *Competitiveness* (0.69) border on the accepted cut-off of 0.70. *Risk Approach* (0.64) obtained the lowest reliability, closely followed by *Conscientiousness* (0.65) and *Ambiguity Acceptance* (0.65). Six of the 78 HPTi items did not fit the Rasch model, all of which underfit the model. Trait *Curiosity* was found not to be unidimensional, while the *Ambiguity Acceptance* scale approached the value at which a scale is considered multidimensional. One item was identified to be threatening the unidimensionality of the *Curiosity* scale based on both the factor loadings of the principal components analysis of the residuals and underfitting the Rasch model. The differential item functioning (DIF) analysis found no item bias between genders, female and male. Eleven items displayed DIF across ethnicities and home language groups. The most severe instance of DIF occurred in trait *Competitiveness*, yet it had only one item experiencing DIF. Trait *Conscientiousness*, however, contained four items experiencing various severities of DIF.

**Contribution:**

This study highlighted the shortcomings of the current HPTi in the South African context through Rasch analysis. The findings illustrate the difficult nature of creating ideal personality instruments in the South African context, thus contributing to the body of knowledge of personality assessments in South Africa.

## Introduction

The use of psychometric testing in decision-making is commonplace in various sectors. Sectors include education, human resources, coaching, forensics, counselling, medical and clinical applications and economic and financial sectors (Arráiz et al., 2016; Bichi, 2016; Coaley, 2010; Foxcroft & Roodt, 2018). Psychometric tools therefore have an importance in various settings globally, as they provide a measurement of psychological constructs not easily observed (Foxcroft & Roodt, 2018). It is also a requirement in South Africa that psychological assessments used in areas of employment show scientific evidence that they are valid and reliable, can be applied fairly and show no bias towards groups (*Employee Equity Act* No. 55 of 1998, Government Gazette, 2014). The High Potential Trait Indicator (HPTi; MacRae & Furnham, 2016) is one such psychometric assessment that is used in South Africa which needs to comply with employment equity (EE) requirements.

## The High Potential Trait Indicator

The HPTi is a self-reporting six-trait personality-based questionnaire with a seven-point Likert-type scale. It was developed in the United Kingdom to identify high performers (MacRae & Furnham, 2016; 2020) and has since been used globally. The six traits are: *Conscientiousness, Adjustment, Curiosity, Risk Approach* (also known as *Courage*), *Ambiguity Acceptance* and *Competitiveness* (MacRae & Furnham, 2016; 2020). The instrument comprises 78 items, 13 per trait, which respondents are required to rate from strongly disagree (1) to strongly agree (7) per item.

In its initial development, the six HPTi scales achieved sufficient internal consistency reliability, with alpha coefficients above 0.70 (MacRae & Furnham, 2020). The initial sample consisted of 779 working professionals across 25 countries (MacRae & Furnham, 2020). Regarding structural validity, MacRae and Furnham (2020) reported structural equation modelling statistics. The comparative fit indices (CFIs) ranged from 0.727 (*Curiosity*) to 0.876 (*Ambiguity Acceptance*). Root mean error of approximation (RMSEA) indices ranged from 0.062 (*Ambiguity Acceptance*) to 0.109 (*Curiosity*). Standard root mean squared indices ranged from 0.047 (*Ambiguity Acceptance*) to 0.078 (*Curiosity*). The HPTi also significantly correlated with various other aspects of certain assessments such as the Hogan Development Survey (HDS; Hogan & Hogan, 2009), NEO Personality Inventory Form S (NEO-PI-R; McCrae & Costa, 1985) and Trait Emotional Intelligence Questionnaire (TEIQue; Petrides, 2009) and demonstrated sufficient predictive validity (MacRae & Furnham, 2016, 2020).

### Definition of the six traits

According to MacRae and Furnham (2020), *Conscientiousness* is a higher-order personality trait in the Five Factor Model. It comprises industriousness, self-control, responsibility, order, traditionalism and virtue. This trait has been found to have a moderate correlation with job success and other job metrics (Barrick et al, 2001; MacRae & Furnham, 2016)

*Adjustment* is described by MacRae and Furnham (2020) as emotional resilience to stress and positive affect and is the inverse of trait *Neuroticism* in the Five Factor Model. Higher levels of *Adjustment* were found to be associated with better teamwork and higher performance, while lower levels were associated with low job satisfaction and subjective well-being (Judge & Locke, 1992; MacRae & Furnham, 2020).

*Curiosity* is synonymous with the openness trait of the Five Factor Model. The trait is characterised as being open to new ideas and experiences, as well as being creative, reflective and innovative (MacRae & Furnham, 2020). *Curiosity* and openness were associated with job satisfaction, trainability and learning outcomes (Barrick et al, 2001; Judge et al, 1999; Linden et al, 2010; MacRae & Furnham, 2020).

*Risk Approach* is defined as how an individual handles challenging, difficult or threatening situations (MacRae & Furnham, 2016). It is the mitigation of negative, threat-based emotions that cause a strong drive to avoid that situation, restricting the potential range of responses to avoidance (MacRae & Furnham, 2020).

*Ambiguity Acceptance* is a measure of how an individual perceives and processes unfamiliarity and that which is not clear (MacRae & Furnham, 2020). Herman et al. (2010) suggest that tolerance for ambiguity involves unfamiliarity, change, challenging perspectives and valuing diversity. High-fliers and senior leadership are thought to require a tolerance for and adaption to ambiguity because of the need to make sense of and incorporate multiple streams of mixed information to make effective decisions (Keenan & McBain, 1979; MacRae & Furnham, 2020; McCall, 1997).

Finally, MacRae and Furnham (2020) describe *Competitiveness* as a dimension that drives self-improvement and the desire for success. In a study of sales performance, Wang and Netemeyer (2002) found competitiveness to be a significant predictor of performance.

## Rasch measurement

Rasch measurement theory (RMT; Rasch, 1960) is mathematically identical to the one-parameter logistic model (1PL) level of item response theory (IRT). Rasch, therefore, is synonymous with 1PL IRT (Finch et al., 2016). However, the two theories developed in separate areas of the world around the same time (Lord & Novick, 1968; Rasch, 1960). A key difference between the two psychometric paradigms is that IRT requires the model to fit the data, whereas RMT prescribes a model which the data must fit (Petrillo et al., 2015). Boone et al. (2014, p. 220) provide an adequate summary of the function of the Rasch model, stating that ‘the Rasch model is a definition of measurement. If persons and items do not fit the model, then those items and persons are not contributing to useful measurement’. Wright and Mok (2004) maintain that the Rasch model is the only model to satisfy the five-model requirements of measurement. These are that the measurement model must:

(a) produce linear measures, (b) overcome missing data, (c) give estimates of precision, (d) have devices for detecting misfitting items/persons, and (e) the parameters of the object being measured and of the measurement instrument must be separable (Wright and Mok 2004, p. 4).

To satisfy the requirements of measurement from a Rasch perspective and psychometric evaluation, the analyses, then, are to evaluate the person reliability, fit to the Rasch model and differential item functioning (DIF) (Bond et al., 2020). Assumptions about the latent traits exist for Rasch analyses (Fan & Bond, 2019). These are that the scales are unidimensional (measure one dimension) and that the items are locally independent (responses to an item do not rely on the response to another item). Tests for unidimensionality and local independence are therefore also necessary in conducting Rasch analyses (Fan & Bond, 2019).

### Fit statistics

Rasch analysis provides two fit statistics for persons and items: infit and outfit statistics. Winsteps (Linacre, 2020a) provides a couple of infit and outfit statistics, namely mean-square (MNSQ), which is an average value of residuals, and z-standardised (ZSTD), which is a *t*-statistic (Boone et al, 2014; Bond et al., 2020).

A reasonable fit statistic range for rating scales, such as Likert-type scales, contains MNSQ values between 0.6 and 1.4 (Wright & Linacre, 1994). On the other hand, MNSQ values less than 0.6 with a ZSTD greater than 2.0 are indicative of an overfitting item and can be interpreted as being more than 40% (1.0 – 0.6 = 0.4) less varied than the Rasch model expects (Bond et al., 2020). A MNSQ value greater than 1.4 with ZSTD greater than 2.0 is indicative of an underfitting item and can be interpreted as being 40% more varied than the Rasch model expects (Bond et al., 2020).

According to Linacre (2020a), outfit is outlier sensitive, and high outfit values tend to be the result of random responses from lower performers. Infit, on the other hand, is information weighted and therefore less influenced by outliers. High infit values are an indication of the items mis-performing and are a greater threat to validity. Linacre (2020a) then suggests that outfit be examined before infit. However, Bond et al. (2020) indicate that deviant infit statistics are more concerning than deviant outfit statistics. Therefore, while outfit will be reported, infit statistics will be the focus, as this statistic is a greater concern to the validity of the scale.

### Person reliability and separation

The reliability of an instrument is its degree of consistency at measuring what it purports to measure (Roodt & De Kock, 2018). A typical measure of a questionnaire’s reliability is internal consistency reliability, traditionally evaluated by Cronbach’s (1951) alpha coefficient.

In Rasch measurement, internal consistency reliability is reported as two metrics: person reliability and person separation (Bond et al., 2020). Rasch reliability statistics indicate the reproducibility of the person ordering or placement (Wright & Masters, 1982). That is, if the same group of respondents took an equivocal test, would they be placed in a similar order based on their measures? Boone et al. (2014) indicate that the person reliability statistics produced by Winsteps are interpreted similarly to traditional reliability indices. Therefore, using conventional guidelines, a person reliability index of 0.7 or higher is considered acceptable (DeVillis, 2017; Kaplan & Saccuzzo, 2017; MacRae & Furnham, 2016; Nunnally, 1978; Pallant, 2020; Yang & Green, 2011). Person separation, on the other hand, is the spread of the respondents on that measure (Bond et al., 2020). A higher separation statistic indicates a larger spread of person measures, with and index of 1.50 regarded as acceptable and 2.00 and 3.00 as good and excellent, respectively (Wright & Masters, 1982).

### Unidimensionality and local independence

Unidimensionality and local independence are two interrelated conditions required for Rasch measurement (Fan & Bond, 2019; Heffernan et al., 2019). Unidimensionality refers to the measurement of a single construct (or latent trait or dimension). For example, the trait *Extraversion* can be considered a single construct. A scale measuring extraversion alone is therefore unidimensional. A scale that measures extraversion and anxiety, then, is multidimensional (measuring more than one dimension). In Rasch measurement, the requirement, then, is that each latent trait be measured one at a time (Fan & Bond, 2019) and is therefore unidimensional. Perfect unidimensionality, however, is not a realistic expectation. Instruments are then required to have a close approximation to unidimensionality. The unidimensionality of an instrument is estimated through a principal component analysis of the residuals (PCAR) (Fan & Bond, 2019), available in software such as Winsteps (Linacre, 2020b). From the PCAR, contrasts with eigenvalues at or greater than 2 indicate the possibility of the scale possessing more than one dimension, whereas contrasts with eigenvalues less than 2 are regarded as insignificant (Bond et al., 2020). Furthermore, items underfitting the Rasch model provide additional concerns to the threat of unidimensionality (Fan & Bond, 2019).

Local independence is the condition in which an individual’s responses to an item is not affected by their response to any other items (Fan & Bond, 2019). For example, an item regarding reading several times a week would affect, or be affected by, an item regarding reading once a week. In such a case, the items are dependent rather than independent and therefore violate the condition of local independence (Fan & Bond, 2019). However, like unidimensionality, it is unrealistic to expect perfect independence. An estimate of the correlation between item residuals is therefore required to determine whether there are items that are significantly dependent on each other (Fan & Bond, 2019; Linacre, 2020a). According to Linacre, positive correlations of 0.7 are the beginning of concern for dependency. Furthermore, items overfitting the Rasch model provide additional concerns to the threat of local independence (Fan & Bond, 2019).

### Differential item functioning

Differential item functioning is an evaluation of how congruently the items of a measure define a construct between certain groups (Boone et al., 2014). In Winsteps (Linacre, 2020b), average measures of the relevant groups (e.g. male and female groups) are presented in logits and are compared against each other. Boone et al. (2014) indicate that DIF may be present in comparisons with a significant *p*-value (*p* < 0.05) of the Rasch–Welch statistic. Linacre (2020a) substantiates that in addition to statistical significance between groups, an effect size ≥ 0.64 is considered moderate to large, whereas between 0.43 and 0.64 is considered slight to moderate. Below 0.43 is considered negligible and insufficient to flag items as having DIF present. A DIF effect size in a Rasch analysis is provided in Winsteps as ‘DIF contrast’ (Linacre, 2020a).

## Methods

### Sample

Table 1 provides a breakdown of the sample. The sample consisted of 1257 respondents who completed the HPTi through the South African subsidiary of Thomas International and had agreed to participate in further research. Slightly more than half of the sample were female (*n* = 684, 54.4%) and the rest male (*n* = 573, 45.6%).

**TABLE 1:** Demographic statistics of the sample.

Variable	*n*	%
Total	1257	-
**Gender**		
Male	573	45.6
Female	684	54.4
**Ethnicity**		
Black	380	30.2
Mixed race	184	14.6
Asian and Indian	106	8.4
White	577	45.9
Other	10	0.8
**Home language**		
English	558	44.4
Afrikaans	370	29.4
Sesotho	48	3.8
isiXhosa	76	6.0
isiZulu	59	4.7
Setswana	49	3.9
Sepedi	47	3.7
Xitsonga	16	1.3
isiNdebele	2	0.2
siSwati	9	0.7
Tshivenda	20	1.6
French	3	0.2
**Provincial location**		
Eastern Cape	80	6.4
Free State	123	9.8
Gauteng	586	46.6
KwaZulu-Natal	112	8.9
Limpopo	13	1.0
Mpumalanga	15	1.2
North West	10	0.8
Northern Cape	5	0.4
Western Cape	313	24.9

Regarding ethnic background, slightly less than half reported as white (*n* = 577, 45.9%). Less than a third reported as black (*n* = 380, 30.2%), followed by mixed race (*n* = 184, 14.6%), then Asian and Indian (*n* = 106, 8.4%). Ten (0.8%) respondents reported other.

When indicating their home language, 558 (44.4%) reported English and 370 (29.4%) indicated Afrikaans as their home language. IsiXhosa was the next most frequently reported home language with 76 (6.0%) respondents, followed by isiZulu (*n* = 59, 5.7%), Setswana (*n* = 49, 3.9%), Sesotho (*n* = 48, 3.8%), Sepedi (*n* = 47, 3.7%), Tshivenda (*n* = 20, 1.6%), Xitsonga (*n* = 16, 1.6%), siSwati (*n* = 9, 0.7%), French (*n* = 3, 0.2%) and isiNdebele (*n* = 2, 0.2%).

Nearly half of the respondents reported Gauteng (*n* = 586, 46.6%) as their residential province. The Western Cape (*n* = 313, 24.9%) was the next most frequently reported residential province, followed by the Free State (*n* = 123, 9.8%), KwaZulu-Natal (*n* = 112, 8.9%), the Eastern Cape (*n* = 80, 6.4%), Mpumalanga (*n* = 15, 1.2%), Limpopo (*n* = 13, 1.0%), North West (*n* = 10, 0.8%) and the Northern Cape (*n* = 5, 0.4%).

Table 2 contains the median, mode, oldest, youngest and range of birth years of the sample. The youngest respondent was born in 1999 and the oldest in 1945. The most commonly occurring year of birth was 1985.

**TABLE 2:** Descriptive statistics of the years of birth of respondents.

Statistics	Year
Median	1982
Mode	1985
Oldest	1945
Youngest	1999
Range	54

### Data collection

Secondary data were obtained from Thomas International Ltd – the intellectual property right holder of the HPTi. The dataset includes the raw data, with scores from strongly disagree (1) to strongly agree (7) of 1257 individuals. The participants completed the HPTi for various purposes, including third-party recruitment and research conducted by Thomas International Ltd. Only data of respondents who completed the HPTi through the South African division of the organisation and had indicated their voluntary participation in further research were obtained. Negatively phrased items were reverse scored.

### Data analysis

The primary data analyses were conducted in Winsteps (Linacre, 2020b) using the Rasch rating scale model. The descriptive statistics were constructed in Microsoft Excel (Microsoft Corporation, Redmond, Washington, United States) and Winsteps (Linacre, 2020b). Each of the six traits were analysed on person fit; descriptive statistics; item reliability and separation; item fit; unidimensionality and local dependence; and DIF.

#### Descriptive statistics

Descriptive statistics were examined using Microsoft Excel to outline the trends in the demographics of the sample (Table 1 and Table 2), and responses of the sample (Table 3). The scale statistics (Table 3) were calculated from the person measures obtained from Winsteps.

**TABLE 3:** Descriptive statistics of person measure scores and reliability indices of Each High Potential Trait Indicator trait.

Trait	Mean	SD	Median	Minimum	Maximum	Skewness	Kurtosis	Alpha	P. Reliability	P. Separation
CN	1.30	0.77	1.16	0.00	4.89	2.06	6.19	0.68	0.65	1.37
AJ	0.82	0.57	0.76	0.63	4.81	1.56	6.58	0.75	0.69	1.49
CU	1.17	0.72	1.06	−0.33	5.47	1.65	5.76	0.75	0.73	1.62
RA	0.61	0.48	0.58	−0.46	3.82	1.43	5.12	0.69	0.64	1.33
AA	−0.07	0.37	−0.10	−1.28	1.30	0.19	0.66	0.68	0.65	1.35
CM	0.06	0.40	0.07	−1.61	1.89	0.24	1.70	0.72	0.69	1.48

SD, standard deviation; AA, Ambiguity Acceptance; AJ, Adjustment; CM, Competitiveness; CN, Conscientiousness; CU, Curiosity; RA, Risk Approach; Alpha, Cronbach’s alpha; P. Reliability, Person Reliability; P. Separation, Person Separation.

#### Person reliability and separation

The person reliability indices of each trait were evaluated to which a person reliability of 0.70 and separation of 1.50 are regarded as sufficiently reliable.

#### Item fit

Misfitting items in the item fit analysis for each HPTi trait were detected and labelled as either underfitting or overfitting the model based on the infit statistics: 0.60 ≥ mean squared (MNSQ) ≥ 1.40 and z-standardised (ZSTD) ≥ |2|.

#### Unidimensionality and local independence

The unidimensionality of each HPTi scale was evaluated through PCAR in Winsteps (Linacre, 2020b). Scales demonstrating contrasts with eigenvalues ≥ 2 are considered to be in violation of unidimensionality (Fan & Bond, 2019). Winsteps (Linacre, 2020b) provides the eigenvalues of more than one contrast. The eigenvalue of the first contrast is provided for each trait (see Table 4, column ‘1c Load’).

**TABLE 4:** Item statistics and fit status.

Item	Measure	1c Load.	IN.MNSQ	IN.ZSTD	OUT.MNSQ	OUT.ZSTD	Fit status
**CN**							
CN01	−0.71	0.03	1.42	5.32	1.41	5.18	Underfit
CN02	−0.32	0.18	0.87	−2.01	0.87	−2.07	Fit
CN03	0.42	−0.24	1.01	0.20	1.15	2.74	Fit
CN04	−1.04	0.54	0.92	−1.14	0.65	−5.27	Fit
CN05	−0.07	0.12	0.96	−0.67	1.06	0.97	Fit
CN06	0.54	−0.14	1.02	0.57	1.20	3.91	Fit
CN07	0.08	0.62	0.84	−3.02	0.91	−1.55	Fit
CN08	−0.35	0.75	0.88	−1.90	0.74	−4.32	Fit
CN09	0.10	−0.04	1.13	2.36	1.26	4.11	Fit
CN10	0.06	−0.16	2.02	9.90	2.14	9.90	Underfit
CN11	−0.19	0.24	0.73	−4.75	0.74	−4.49	Fit
CN12	0.90	−0.33	1.01	0.20	1.15	3.41	Fit
CN13	0.57	−0.37	1.14	3.28	1.19	3.82	Fit
**AJ**							
AJ01	0.26	−0.45	0.70	−8.90	0.72	−7.28	Fit
AJ02	−0.73	−0.47	1.29	4.33	1.07	1.14	Fit
AJ03	−0.75	−0.41	1.24	3.51	1.14	2.12	Fit
AJ04	0.59	0.46	1.08	2.27	1.14	3.61	Fit
AJ05	0.04	−0.31	0.89	−2.67	1.00	0.09	Fit
AJ06	−0.32	−0.31	1.42	7.19	1.31	5.09	Underfit
AJ07	0.17	0.45	1.20	4.87	1.26	5.36	Fit
AJ08	−0.42	−0.06	1.00	0.00	0.94	−1.06	Fit
AJ09	0.52	−0.43	0.89	−3.25	1.01	0.21	Fit
AJ10	0.39	0.05	1.19	4.82	1.30	6.65	Fit
AJ11	−0.07	0.16	0.96	−0.96	1.08	1.53	Fit
AJ12	0.41	0.56	1.19	4.96	1.35	7.84	Fit
AJ13	−0.08	0.10	0.90	−2.40	0.87	−2.72	Fit
**CU**							
CU01	−0.05	0.21	0.81	−4.35	0.84	−3.59	Fit
CU02	−0.52	0.45	0.73	−5.74	0.69	−6.53	Fit
CU03	0.81	−0.52	1.10	2.62	1.21	5.06	Fit
CU04	−0.93	0.41	0.92	−1.54	0.86	−2.47	Fit
CU05	−0.86	0.64	0.92	−1.47	0.85	−2.71	Fit
CU06	0.46	0.09	1.24	5.62	1.25	5.53	Fit
CU07	−0.73	0.58	0.80	−3.97	0.74	−5.08	Fit
CU08	0.14	0.09	1.18	3.93	1.33	6.57	Fit
CU09	−0.20	0.09	1.03	0.54	1.07	1.40	Fit
CU10	0.29	−0.12	0.97	−0.66	1.13	2.91	Fit
CU11	1.06	−0.44	1.25	6.47	1.30	7.32	Fit
CU12	−0.39	0.57	0.80	−4.20	0.81	−4.01	Fit
CU13	0.93	−0.50	1.32	7.95	1.45	9.90	Fit
**RA**							
RA01	0.31	−0.26	0.93	−2.07	1.03	0.69	Fit
RA02	0.48	−0.60	1.31	8.20	1.39	9.35	Fit
RA03	−0.13	0.62	0.81	−4.60	0.80	−4.45	Fit
RA04	0.59	0.38	0.86	−4.48	0.95	−1.37	Fit
RA05	0.37	0.19	0.82	−5.29	0.87	−3.42	Fit
RA06	−0.04	−0.18	1.19	4.15	1.31	6.21	Fit
RA07	−0.42	0.45	0.86	−2.89	0.88	−2.35	Fit
RA08	−0.18	−0.22	1.28	5.63	1.36	6.77	Fit
RA09	0.22	−0.19	1.16	4.07	1.32	7.09	Fit
RA10	−0.70	0.48	0.87	−2.31	0.79	−4.05	Fit
RA11	−0.22	−0.14	0.86	−3.22	0.89	−2.22	Fit
RA12	−0.31	0.34	0.88	−2.57	1.03	0.54	Fit
RA13	0.03	−0.15	1.44	9.37	1.62	9.90	Underfit
**AA**							
AA01	−0.60	−0.10	1.48	9.90	1.44	9.15	Underfit
AA02	0.99	0.47	1.06	1.18	1.03	0.52	Fit
AA03	−0.42	−0.11	1.05	1.36	1.04	1.02	Fit
AA04	0.06	0.12	0.82	−5.80	0.82	−5.55	Fit
AA05	0.17	0.07	1.25	6.86	1.28	7.35	Fit
AA06	−0.30	−0.26	0.96	−1.23	0.94	−1.74	Fit
AA07	0.09	0.31	0.75	−8.32	0.76	−7.64	Fit
AA08	−0.77	−0.63	0.96	−0.97	0.98	−0.33	Fit
AA09	0.32	−0.07	0.93	−1.96	0.95	−1.27	Fit
AA10	0.39	0.54	0.80	−5.83	0.79	−5.74	Fit
AA11	0.08	−0.07	1.08	2.45	1.14	3.74	Fit
AA12	0.56	0.68	0.69	−8.81	0.66	−8.98	Fit
AA13	−0.58	−0.54	1.39	8.88	1.54	9.90	Underfit
**CM**							
CM01	−0.14	−0.24	0.84	−4.65	0.83	−4.91	Fit
CM02	−2.04	−0.30	1.34	4.57	1.25	3.49	Fit
CM03	0.11	−0.21	0.78	−6.94	0.83	−5.21	Fit
CM04	0.26	−0.39	0.88	−3.86	0.90	−2.98	Fit
CM05	0.01	−0.52	1.01	0.17	1.00	0.15	Fit
CM06	0.08	−0.18	0.99	−0.44	1.05	1.47	Fit
CM07	0.21	0.50	1.07	2.06	1.14	3.85	Fit
CM08	0.16	0.35	1.17	4.92	1.20	5.31	Fit
CM09	0.64	0.60	0.94	−1.64	0.98	−0.41	Fit
CM10	0.42	0.15	1.14	3.90	1.15	3.92	Fit
CM11	0.26	0.37	0.98	−0.69	0.99	−0.17	Fit
CM12	−0.38	−0.21	0.90	−2.55	0.87	−3.38	Fit
CM13	0.42	−0.12	1.32	8.54	1.34	8.45	Fit

AA, Ambiguity Acceptance; AJ, Adjustment; CM, Competitiveness; CN, Conscientiousness; CU, Curiosity; RA, Risk Approach; 1c Load., first contrast loading.

Evidence for local independence of each HPTi scale items was based on correlations between item residuals. From these estimates, positive correlations between items of 0.7 or higher are considered to be in violation of local independence (Fan & Bond, 2019).

#### Differential item functioning

The DIF was analysed across the items of each HPTi trait and between the relevant subgroups by means of a significant difference in the Rasch–Welch statistic and a sufficiently large DIF contrast. A *p*-value less than 0.05 in Winsteps indicates a significant difference, and an effect size of at least 0.43, as indicated by DIF contrast in Winsteps, is considered large enough.

The subgroups for the DIF analysis are gender, ethnicity and language. However, because of the differences in sample sizes between the European languages (English and Afrikaans) and African languages (Sesotho, isiXhosa, isiZulu, Setswana, Sepedi, Xitsonga, isiNdebele and siSwati), the African languages were collapsed to form the group ‘African Languages’. The resultant language groups are thus African languages (*n* = 326, 26.2%), Afrikaans (*n* = 370, 29.4%) and English (*n* = 558, 44.4%). This is not intended to reduce the differences in the African languages and may warrant further investigation with larger samples in the individual African languages.

### Ethical considerations

The secondary data obtained from Thomas International Ltd (thomas.co) contained the anonymised responses of individuals who completed the HPTi and indicated their voluntary participation in further research. Respondents were presented with the opportunity to indicate their voluntary participation in further research after completing the HPTi. Ethical approval was obtained from the Psychology Research and Ethics Committee at the University of Pretoria (reference number: HUM037/0720).

## Results

### Reliability

The reliability indices ranged from adequate to inadequate (see Table 3). *Curiosity* obtained the highest reliability indices with a person reliability of 0.73 and separation of 1.62. *Risk Approach* (0.64, 1.33) had the lowest reliability indices, followed closely by *Ambiguity Acceptance* (0.65, 1.35), *Conscientiousness* (0.65, 1.37), then *Competitiveness* (0.69, 1.48) and *Adjustment* (0.69, 1.49).

### Item fit

Outfit and infit statistics were evaluated for the items of the HPTi traits, with precedence given to infit. The infit and outfit statistics of the items can be viewed in Table 4.

*Conscientiousness* contained two misfitting items: CN01 and CN10 underfit the model (IN.MNSQ = 1.42 and 2.02, IN.ZSTD = 5.32 and 9.90, respectively). *Adjustment* had one misfitting item, where AJ06 underfit the model (IN.MNSQ = 1.42, IN.ZSTD = 7.19). *Risk Approach* contained one underfitting item: RA13 (IN.MNSQ = 1.44, IN.ZSTD = 9.37). *Ambiguity Acceptance* had two misfitting items: AA01 (IN.MNSQ = 1.48, IN.ZSTD = 9.90) and AA13 (IN.MNSQ = 1.39, IN.ZSTD = 8.88) underfit the model.

### Unidimensionality and local independence

The unidimensionality of each scale was examined through PCAR. The item loadings of the first contrast can be seen in Table 4 as ‘1c Load.’. The results revealed that *Curiosity* had the highest first contrast eigenvalue (λ = 2.24), followed by *Ambiguity Acceptance* (λ = 1.87), *Adjustment* (λ = 1.75), *Conscientiousness* (λ = 1.70), *Risk Approach* (λ = 1.70) and *Competitiveness* (λ = 1.61).

The largest standardised residual correlations were analysed to evaluate the local independence of the items of the scale. No item pairs were found to be above the correlation of 0.70, indicating that none of the items of the scales are in violation of local independence.

### Differential item functioning

#### Gender

The analysis of DIF on gender revealed no items in concern across all HPTi scales. While levels of significance were detected, the significant items are not described because the DIF effect sizes of these items were negligible, indicating no practical significance. Table 5 provides the items with significant *p*-values for DIF between gender groups.

**TABLE 5:** The DIF between gender groups.

Item	Mean (S.E.)		*p*-value	DIF contrast	Effect size
	Female	Male			
**CN**					
CN01	−0.81 (0.06)	−0.60 (0.06)	0.02	−0.21	Negligible
CN02	−0.40 (0.05)	−0.22 (0.05)	0.01	−0.19	Negligible
CN05	0.00 (0.04)	−0.16 (0.05)	0.01	0.15	Negligible
CN08	−0.44 (0.05)	−0.25 (0.05)	0.01	−0.19	Negligible
CN10	0.12 (.04)	−0.01 (0.04)	0.02	0.13	Negligible
CN11	−0.11 (0.04)	−0.28 (0.05)	0.01	0.18	Negligible
**AJ**					
AJ03	−0.05 (0.03)	0.15 (0.03)	0.00	−0.21	Negligible
AJ05	−0.26 (0.03)	−0.40 (0.04)	0.01	0.15	Negligible
AJ06	−0.49 (0.04)	−0.33 (0.04)	0.00	−0.16	Negligible
AJ08	0.58 (0.03)	0.45 (0.03)	0.00	0.13	Negligible
AJ09	0.43 (0.03)	0.33 (0.03)	0.02	0.10	Negligible
**CU**					
CU01	0.01 (0.04)	−0.12 (0.04)	0.02	0.14	Negligible
CU02	−0.45 (0.04)	−0.61 (0.05)	0.02	0.16	Negligible
**RA**					
RA12	−0.35 (0.03)	−0.25 (0.04)	0.04	−0.11	Negligible
RA13	0.07 (0.03)	−0.02 (0.03)	0.05	0.09	Negligible
**AA**					
AA01	−0.64 (0.03)	−0.54 (0.03)	0.02	−0.09	Negligible
AA07	0.14 (0.03)	0.04 (0.03)	0.01	0.10	Negligible
**CM**					
CM03	0.06 (0.02)	0.17 (0.03)	0.00	−0.11	Negligible
CM04	0.31 (0.03)	0.21 (0.03)	0.01	0.10	Negligible
CM05	0.10 (0.02)	−0.11 (0.03)	0.00	0.21	Negligible
CM06	0.00 (0.03)	0.17 (0.03)	0.00	−0.16	Negligible
CM07	0.16 (0.02)	0.27 (0.03)	0.00	−0.11	Negligible
CM08	0.22 (0.02)	0.08 (0.03)	0.00	0.14	Negligible

DIF, differential item functioning; S.E., standard error; AA, Ambiguity Acceptance; AJ, Adjustment; CM, Competitiveness; CN, Conscientiousness; CU, Curiosity; RA, Risk Approach.

#### Ethnicity

The presence of DIF was evaluated between ethnicities. Table 6 displays the items across the traits with statistically significant and sufficiently large DIF effect sizes. Other instances of statistically significant differences were present; however, upon the evaluation of their DIF contrasts, their effect sizes were found to be negligible and not included. The *Adjustment* scale had no instances exhibiting DIF between ethnicities.

**TABLE 6:** Differential item functioning between ethnicity groups.

Item	Group 1	Mean (S.E.)	Group 2	Mean (S.E.)	*p*	DIF contrast	Effect size
**CN**							
CN04	A/I	−1.41 (0.22)	W	−0.81 (0.07)	0.00	−0.59	Slight to moderate
CN04	B	−1.45 (0.12)	C	−0.98 (0.13)	0.03	−0.47	Slight to moderate
CN04	B	−1.45 (0.12)	W	−0.81 (0.07)	0.00	−0.63	Moderate to large
CN08	A/I	−0.62 (0.15)	W	−0.19 (0.05)	0.01	−0.43	Slight to moderate
**CU**							
CU02	B	−0.87 (0.08)	W	−0.35 (0.04)	0.00	−0.52	Slight to moderate
CU04	A/I	−1.23 (0.16)	C	−0.73 (0.09)	0.01	−0.51	Slight to moderate
CU04	A/I	−1.23 (0.16)	W	−0.77 (0.05)	0.01	−0.47	Slight to moderate
CU04	B	−1.38 (0.10)	C	−0.73 (0.09)	0.00	−0.65	Moderate to large
CU04	B	−1.38 (0.10)	W	−0.77 (0.05)	0.00	−0.61	Slight to moderate
CU06	A/I	0.67 (0.08)	B	0.22 (0.05)	0.00	0.45	Slight to moderate
CU13	A/I	0.76 (0.08)	B	1.29 (0.04)	0.00	−0.53	Slight to moderate
CU13	B	1.29 (0.04)	C	0.85 (0.06)	0.00	0.44	Slight to moderate
CU13	B	1.29 (0.04)	W	0.76 (0.03)	0.00	0.53	Slight to moderate
**RA**							
RA13	C	−0.39 (0.07)	B	0.06 (0.04)	0.00	−0.45	Slight to moderate
RA13	C	−0.39 (0.07)	W	0.13 (0.03)	0.00	−0.51	Slight to moderate
**AA**							
AA02	B	1.44 (0.07)	A/I	1.02 (0.09)	0.00	0.42	Slight to moderate
AA02	B	1.44 (0.07)	C	0.96 (0.07)	0.00	0.48	Slight to moderate
AA02	B	1.44 (0.07)	W	0.82 (0.03)	0.00	0.62	Slight to moderate
AA09	B	0.08 (0.03)	W	0.50 (0.03)	0.00	−0.42	Slight to moderate
**CM**							
CM02	A/I	−2.46 (0.20)	C	−1.98 (0.12)	0.04	−0.48	Slight to moderate
CM02	A/I	−2.46 (0.20)	W	−1.73 (0.06)	0.00	−0.73	Moderate to large
CM02	B	−2.58 (0.11)	C	−1.98 (0.12)	0.00	−0.60	Slight to moderate
CM02	B	−2.58 (0.11)	W	−1.73 (0.06)	0.00	−0.85	Moderate to large

DIF, differential item functioning; S.E., standard error; AA, Ambiguity Acceptance; AJ, Adjustment; CM, Competitiveness; CN, Conscientiousness; CU, Curiosity; RA, Risk Approach; A/I, Asian and/or Indian; B, black African; C, mixed race; W, white.

Four instances where DIF may be present in the *Conscientiousness* scale were identified. These instances spanned across two items: CN04 and CN08. Item CN04 had the most instances in which DIF may be present between ethnicities, with three of the four occurrences. Item CN04 also had the greatest effect size of the four instances (DIF contrast = –0.63) between the black African and white groups.

Nine instances of possible DIF were identified in the *Curiosity* scale. Instances occurred in items CU02, CU04, CU06, CU13. Items CU04 had the highest number of DIF instances and the instance with the largest effect size (DIF contrast = –0.65) between the black African and mixed-race groups, followed closely between the black African and white groups (DIF contrast = –0.61).

Two DIF instances were revealed across one *Risk Approach* item, RA13. The largest instance, in terms of effect size, was between the mixed race and white ethnic groups with a moderate to large effect (DIF contrast = –0.51).

*Ambiguity Acceptance* had four instances identified across two items. Item AA02 had the largest effect size (DIF contrast = 0.62) between the black African and white groups.

*Competitiveness* had four instances found across one item. Item CM02 had the largest effect size of all HPTi items (DIF contrast = –0.85) between the black African and white groups.

#### First-language groups

The potential presence of DIF was then evaluated between first-language groups. Table 7 displays the items across the traits with statistically significant and sufficiently large effect sizes. Other instances of statistically significant differences were present; however, upon the evaluation of their DIF contrasts their effect sizes were found to be negligible and not included. *Adjustment* and *Risk Approach* were found not to have items experiencing DIF.

**TABLE 7:** Differential item functioning between language groups.

Item	Group 1	Mean (S.E.)	Group 2	Mean (S.E.)	*p*-value	DIF contrast	Effect size
**CN**							
CN02	Eng	−0.44 (0.06)	AL	−0.04 (0.06)	0.00	−0.41	Approaching slight
CN04	Afr	−0.93 (0.09)	AL	−1.42 (0.13)	0.00	0.48	Slight to moderate
CN04	Eng	−0.93 (0.07)	AL	−1.42 (0.13)	0.00	0.48	Slight to moderate
**CU**							
CU02	Afr	−0.37 (0.06)	AL	−0.88 (0.08)	0.00	0.51	Slight to moderate
CU04	Afr	−0.83 (0.07)	AL	−1.30 (0.10)	0.00	0.48	Slight to moderate
CU04	Eng	−0.84 (0.06)	AL	−1.30 (0.10)	0.00	0.47	Slight to moderate
CU13	Afr	0.87 (0.04)	AL	1.32 (0.04)	0.00	−0.45	Slight to moderate
CU13	Eng	0.75 (0.03)	AL	1.32 (0.04)	0.00	−0.57	Slight to moderate
**AA**							
AA02	Afr	0.92 (0.04)	AL	1.44 (0.07)	0.00	−0.52	Slight to moderate
AA02	Eng	0.87 (0.04)	AL	1.44 (0.07)	0.00	−0.57	Slight to moderate
**CM**							
CM02	Afr	−1.80 (0.08)	AL	−2.51 (0.12)	0.00	0.71	Moderate to large
CM02	Eng	−1.98 (0.07)	AL	−2.51 (0.12)	0.00	0.53	Slight to moderate

DIF, differential item functioning; S.E., standard error; AA, Ambiguity Acceptance; AJ, Adjustment; CM, Competitiveness; CN, Conscientiousness; CU, Curiosity; RA, Risk Approach; AL, African Languages; Afr, Afrikaans; Eng, English.

Three instances of DIF were detected across two *Conscientiousness* items, CN02 and CN04, of which the instance with the largest effect size occurred in item CN04 between the African languages and Afrikaans groups with slight to moderate effect (DIF contrast = 0.50).

*Curiosity* had five instances identified, all of which were slight to moderate and between the African languages group and either English or Afrikaans groups. The largest effect size was found to be in item CU13 between the English-speaking group and African language–speaking group (DIF = –0.57).

One item had been identified with two instances of potential DIF in the *Ambiguity Acceptance* scale. Item AA02 had a slight to moderate effect size between the Afrikaans and African languages groups (DIF contrast = –0.52) and English and African languages groups (DIF contrast = –0.57).

The *Competitiveness* trait also had two instances across one item. Item CM02 had a moderate to large effect size between the Afrikaans and African languages groups (DIF contrast = 0.71) and a slight to moderate effect size between the English and African languages groups (DIF contrast = 0.53).

## Discussion

### Reliability

This study set out to evaluate the psychometric properties of the HPTi, a personality assessment. The psychometric properties were evaluated through Rasch analysis, namely person reliability and separation, fit to the Rasch model and the Rasch version of DIF.

The reliability indices of five of the six HPTi scales would not be considered reliable against the widely accepted minimum standards. The five scales are *Adjustment* (0.69), *Competitiveness* (0.69), *Conscientiousness* (0.65), *Ambiguity Acceptance* (0.65) and *Risk Approach* (0.64), of which *Adjustment* and *Competitiveness* bordered on the minimum value required to be regarded as being reliable. When evaluating other personality-based psychological assessments in the South African context, de Bruin et al.’s (2022) evaluation of the Basic Traits Inventory revealed reliability indices (Cronbach’s alpha) ranging from 0.87 (*Openness*) to 0.94 (*Conscientiousness*) in the adult sample. Similarly, the Myers–Briggs Type Indicator® (MBTI®; Myers et al., 1998) obtained high alphas ranging from 0.88 (both *Sensing–Intuition* and *Thinking–Feeling*) to 0.91 (both *Extraversion–Introversion* and *Judging–Perceiving*. The Rasch person reliability of the Judging–Perceiving dichotomy was 0.83, with the rest being 0.84 (Van Zyl & Taylor, 2012). The South African Personality Inventory (SAPI; Fetvadjiev et al., 2015) achieved mean alphas from 0.71 (*Social Relation – Negative*) to 0.81 (*Social Relation – Positive*), although subscales had alphas as low as 0.61 (*Deceitfulness*). When re-evaluating the SAPI, Morton et al. (2018) obtained alphas ranging from 0.61 (*Neuroticism*) to 0.88 (*Social Relation* – *Positive*). Hill et al. (2021) evaluated the psychometric properties of the Tshivenda and Southern Sotho versions of the SAPI. The Tshivenda version obtained mean alphas ranging from 0.61 (*Extraversion*) to 0.72 (*Social Relation – Positive*). The Southern Sotho version obtained alphas ranging from 0.50 (*Extraversion*) to 0.77 (*Social Relation – Negative*). Boshoff and Laher (2015) reviewed the utility of the NEO-PI-3 (McCrae et al., 2005) in the South African context. They found reliability coefficients ranging from 0.61 (*Agreeableness*) to 0.79 (*Extraversion*) for the domains of the NEO-PI-3. Thus, the reliability findings of the HPTi subscales are neither irregular nor the worst in the South African context but can certainly be improved upon. Person reliability, according to Linacre (2020a), is largely dependent on the dispersion of the characteristics of the sample – in other words, a sample with varying degrees of the trait being measured – the length of the instrument, the number of response options per item and the targeting of the sample and items. Some traits’ reliability indices could potentially be impacted by the inadequate targeting, evident in the large differences between the average person scores in traits *Conscientiousness, Adjustment, Curiosity* and *Risk Approach* and the constrained item measure average of zero. On the matter of targeting, Boone et al. (2014) recommend the revision of the difficulty of the items accordingly, making them either more or less difficult to endorse. On the other hand, the scales that appear well targeted – *Ambiguity Acceptance* and *Competitiveness* – may have their respective reliability indices impacted by the inability to adequately separate the higher scorers on the trait from those who have lower person measures of that trait, resulting in a lower person separation index and therefore reliability index. Given the results, it may be difficult to defend the reliability of the HPTi subscales in the South African context, with *Curiosity* being the most defensible.

### Item fit

Item fit statistics evaluated how well the items of each HPTi trait conformed to the Rasch model. Fit mean-squared statistics greater than 1.40 indicate that the item is 40% less predictable (more varied) than the model. The same statistic under 0.60 indicates that the item is 40% more predictable (less varied) than the model expects (Bond et al., 2020). Six of the 78 HPTi items (7.7%) underfit the Rasch model: *Conscientiousness* and *Ambiguity Acceptance* with two (15.4%) items each, *Adjustment* and *Risk Approach* with one (7.7%) item each, while *Curiosity* and *Competitiveness* had no items underfitting the model. *Curiosity*, however, had one item that bordered on the underfitting criterion of 1.40, item CU13. In contrast, the evaluation of the MBTI® Form M in the South African context had no items across all four dichotomous dimensions overfitting or underfitting the model at the criteria employed in this study (Van Zyl & Taylor, 2012). According to Tennant and Conaghan (2007), however, fit to the Rasch model can be influenced by item bias such as DIF.

### Differential item functioning

The DIF is an evaluation of how congruently the items of a measure define a construct between certain groups (Boone, et al., 2014). It is especially important in cross-cultural settings (Tennant et al., 2004).

Across all scales, while statistically significant DIF was found between gender groups, the findings were not practically significant, as measured by DIF contrast (Linacre, 2020a). This suggests that the HPTi scales do not contain items with definitions that are interpreted differently between men and women. Therefore, none of the items of the HPTi could be considered biased towards either men or women. In contrast, Van Zyl and Taylor (2012) found 12 (13%) of the 93 items with a DIF contrast above 0.43 (slight to moderate effect; Linacre, 2020a) when evaluating DIF between gender, two (2%) of which were above 0.64 (moderate to large effect; Linacre, 2020a).

Apart from the *Adjustment* scale, DIF was discovered in several items in the ethnic groups and first-language groups comparisons. The severity varied across the scales. Between ethnic or racial groups, the largest and second largest exhibitions of DIF occurred in item CM02 in trait *Competitiveness* between the black group and white group (–0.85) and the Asian and Indian group and mixed-race group (-0.73). Most instances of DIF involved the black African group. Similarly, most instances of DIF between language groups involved the African languages group. The DIF between black and white ethnic groups was also found in Van Zyl and Taylor (2012) and between African languages and both English and Afrikaans in Grobler and De Beer’s (2015) evaluation of the Basic Traits Inventory. The findings of the item-level bias are not dissimilar to historic findings of personality questionnaires in the South African context, in which bias between African and European ethnic groups and language groups is usually found and recommended for further investigation (Abrahams & Mauer, 1999; Spence, 1982; Taylor & Boeyens, 1991). It may therefore be required that the relevant HPTi items be re-examined in the South African context to reduce the item-level bias.

## Limitations

This study is not without limitations. Firstly, with respect to the methodology, is the use of secondary data obtained from an organisation whose use in psychometric tools is largely in the recruitment sector (thomas.co, n.d.). This falls into the disadvantage of secondary data research expressed by Boslaugh (2007), in which secondary data are often collected for purposes other than that of the research question using the secondary data. Secondly, the limitation inherent in self-reporting personality assessments, especially used in decision-making, is one in which respondents may respond in a way that distorts or misrepresents them (Coaley, 2010). It is therefore not unimaginable to have obtained data with some responses skewed towards the purposes the HPTi was originally administered for, such as applications to employment. To address these two limitations, further research is encouraged in which respondents are randomly selected, the administration is standardised and the purpose of completing the assessment is exclusively for research and not for, say, the application process for employment.

## Conclusion

The study aimed to evaluate the psychometric properties of the six personality subscales of the HPTi through Rasch analysis and in the South African context. The core properties in question were reliability, fit to the Rasch model and DIF. The results indicate that while some subscales have some redeeming qualities, all subscales have their shortcomings and could be improved on for use in the South African environment. Similar shortcomings have been acknowledged historically and found in more recent evaluations of personality instruments in South Africa. Two other personality instruments, using similar evaluation techniques, achieved high reliability and good fit to the Rasch model but still experienced DIF in certain items between either ethnicity or home language (Grobler & De Beer, 2015; Van Zyl & Taylor, 2012). This illustrates the difficult, but not impossible, nature of creating an ideal personality instrument in the South African context, thus contributing to the wider body of knowledge of personality assessments in South Africa, while simultaneously recommending further improvements upon an instrument used widely in the country.
